# Associations of elevated cardiac biomarkers with hyperuricemia and mortality in US adults without prevalent cardiovascular disease

**DOI:** 10.3389/fendo.2024.1432200

**Published:** 2024-12-05

**Authors:** Haitao Xie, Le Shen, Peng Yu, Shi Wang, Tong Sun, Xueqian Liu, Mengxi Wang, Li Qian, Jiayi Hua, Nan Chen, Xiaohu Chen, Shuhua Tang

**Affiliations:** ^1^ First Clinical Medical College, Nanjing University of Chinese Medicine, Nanjing, China; ^2^ Department of Cardiology, Affiliated Hospital of Nanjing University of Chinese Medicine, Nanjing, China; ^3^ Department of Cardiology, Jiangsu Province Hospital of Chinese Medicine, Nanjing, China; ^4^ First Clinical Medical College, Nanjing Medical University, Nanjing, China

**Keywords:** without known cardiovascular disease, cardiac biomarkers, hyperuricemia, prevalence, risk of mortality, population study

## Abstract

**Background:**

NT-proBNP (N-terminal pro-B-type natriuretic peptide), high-sensitivity cardiac troponin T (hs-troponin T), and high-sensitivity cardiac troponin I (hs-troponin I) have been widely recognized as significant cardiac biomarkers, and are increasingly being recommended for early risk identification in cardiovascular high-risk populations. The aim of our study was to evaluate the prevalence of elevated cardiac biomarkers (NT-proBNP, hs-troponin T, hs-troponin I) and their association with the risk of hyperuricemia in the general US adults without known cardiovascular disease. We further studied whether elevated cardiac biomarkers are associated with an increased risk of all-cause and cardiovascular mortality in individuals with or without hyperuricemia.

**Methods:**

The study population came from the adults (age ≥20y) without prevalent cardiovascular disease in NHANES (National Health and Nutrition Examination Survey) 1999 to 2004. We evaluated the prevalence of elevated cardiac biomarkers among adults with or without hyperuricemia, and conducted a comprehensive multivariate logistic regression analysis to ascertain the association between elevated cardiac biomarkers and hyperuricemia risk. Multivariate Cox regression model and Kaplan-Meier curve, risk competition model and Cumulative Incidence Function(CIF) were used respectively to examine the associations between elevated cardiac biomarkers with all-cause and cardiovascular mortality.

**Results:**

In general US adults without known cardiovascular disease, the prevalence of hyperuricemia was 16.35%. The age-adjustd prevalence of elevated NT-proBNP (≥125 pg/mL), hs-troponin T (≥6 ng/L), and hs-troponin I (male ≥6, female ≥4 ng/L) was 16.70%, 49.80%, and 11.91%, respectively, among adults with hyperuricemia. Adjusted multivariable logistic regression analysis revealed a statistically significant association between elevated levels of NT-proBNP, hs-troponin T, and hs-troponin I and hyperuricemia, and different clinical categories observed grade differences on the same cardiac biomarker. Elevated NT-proBNP, hs-troponinT and hs-troponinI were each significantly positively associated with the cumulative incidence of all-cause and cardiovascular mortality in adults with or without hyperuricemia. Compared to those with elevated cardiac biomarkers only, adults with hyperuricemia and elevated cardiac biomarkers faced the highest risk of all-cause and cardiovascular mortality.

**Conclusions:**

Our study identified that elevated cardiac biomarkers pose a high burden on hyperuricemia risk in the general population without known cardiovascular disease, and further provides important information on long-term mortality risk in these populations. Routine testing of cardiac biomarkers may be useful for early risk identification and prognostic assessment in adults with hyperuricemia.

## Introduction

Hyperuricemia, a common chronic disease resulting from disturbances in purine metabolism or disorders in the excretion of uric acid, which can induce the accumulation of uric acid crystals in joints and other tissues, giving rise to numerous complications such as gout, uric acid nephropathy, and metabolic syndrome (1, 2). Numerous epidemiological studies provide supports for the increased relative risk of hypertension and diabetes caused by hyperuricemia (3–6). According to the European guidelines for arterial hypertension (7), uric acid evaluation is also recommended as an independent risk factor for cardiovascular disease.

In the field of clinical diagnosis and treatment, more and more evidence advocates for the utilization of NT-proBNP (N-terminal pro-B-type natriuretic peptide), high-sensitivity cardiac troponin T (hs-troponin T), and high-sensitivity cardiac troponin I (hs-troponin I) in early risk identification in cardiovascular high-risk populations (8–13). These biomarkers, widely acknowledged for their significance in cardiovascular health assessment, hold substantial value in the classification of risk levels and assessment of prognosis. In contrast to previous detection of cardiac biomarkers only in cases of myocardial injury, the application of high-sensitivity measurement techniques has significantly improved the sensitivity of detecting cardiac biomarkers. Consequently, routine screening tests for cardiac biomarkers can now be conducted even in the general population without any prior history of cardiovascular disease.

Given the extensive research confirming the association between hyperuricemia and increased cardiovascular risk (14, 15), it becomes crucial to acquire a comprehensive understanding and screen of the cardiovascular burden and long-term mortality risk among individuals afflicted with hyperuricemia. However, existing research findings are predominantly based on high-risk groups with cardiovascular disease or other complications, and lack comprehensive data representative of a larger population. In contrast, the association between hyperuricemia and cardiac biomarkers remains unconfirmed in the general population without known cardiovascular disease. Moreover, there is a lack of studies examining the utility of cardiac biomarkers to identify cardiovascular disease burden and long-term adverse outcomes in individuals with hyperuricemia in the general population.

The aim of our research was to evaluate the prevalence of elevated cardiac biomarkers (NT-proBNP, hs-troponin T, hs-troponin I) and their correlation with the risk of hyperuricemia in the general US adults without known cardiovascular disease. Additionally, we further studied whether elevated cardiac biomarkers are associated with an increased risk of all-cause and cardiovascular mortality in individuals with or without hyperuricemia.

## Methods

### Study design and participants

**Figure 1:**
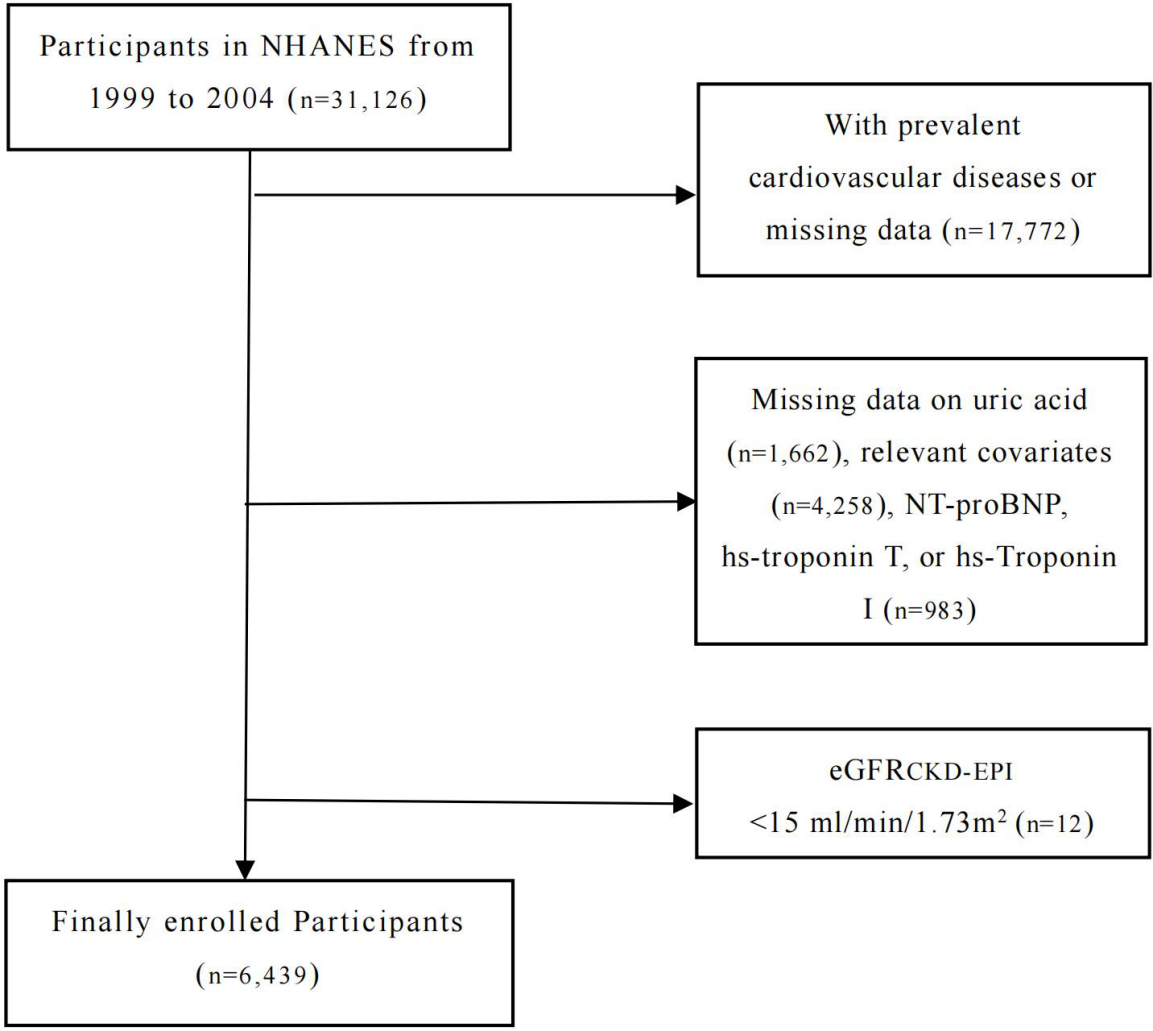
Study design and exclusion information fowchart.

Data for this study were obtained from the National Health and Nutrition Examination Survey (NHANES), a national cross-sectional survey in the US. NHANES aims to collect information from the non-institutionalized civilian population and provide representative samples that reflect the health and nutritional status of the general population. As part of its sampling design, NHANES uses a complex, stratified, and multistage method, combined with standardized personal interviews and mobile physical examinations. All participants obtained written informed consent after receiving approval from both the Centers for Disease Control and Prevention (CDC) and the National Center for Health Statistics (NCHS). In this study, we investigated the associations between the levels of NT-proBNP, hs-Troponin T, and hs-Troponin I and the prevalence of hyperuricemia in adults (≥20 years old) enrolled in the NHANES between 1999 and 2004. Individuals with known cardiovascular disease or missing data (defined as a self-reported coronary artery disease, heart attack, angina, stroke, or heart failure, n=17,772) were excluded, as well as those with missing data on uric acid (n=1,662), relevant covariates (n=4,258), NT-proBNP, hs-troponin T, or hs-Troponin I (n=983). Further, participants with severe renal failure (eGFR <15 ml/min/1.73m^2^, n=12) were also excluded. A total of 6,439 participants were included in our final analysis. The study design and exclusion details are shown in the flowchart (**Figure 1**).

### Measurement of cardiac biomarkers

Between 2018 and 2020, researchers from the Johns Hopkins Bloomberg School of Public Health conducted cardiac biomarker measurements (including NT-proBNP, high-sensitivity troponin T, and high-sensitivity troponin I) on stored venous blood samples from NHANES 1999-2004 at the University of Maryland School of Medicine. Hence, the data on cardiac biomarkers is currently applicable to the period of 1999-2004 (16, 17). NT-proBNP (Roche diagnosis) in serum was measured on the Roche Cobas e601 autoanalyzer. The lower and upper detection limits were 5 pg/mL and 35000 pg/mL, respectively, with coefficients of variation (CV) of 3.1% (low, 46 pg/mL) and 2.7% (high, 32805 pg/mL). Hs-Troponin T in serum was measured by Roche Cobas e601 using Elecsys reagents. The limit of detection (LoD) was 3 ng/L, and CV: 3.1% [26.12-31.28 ng/L] and 2.0% [2004.5-2215 ng/L]. Hs-Troponin I in serum was measured using Abbott ARCHITECT i2000SR, had a LoD of 1.7 ng/L and CV of 6.4% (8.12-16.01 ng/L), 4.5% (27.69-55.38 ng/L), 3.5% (169.1-314.1 ng/L), and 6.7% (2758-4444 ng/L).

### Cardiac biomarkers stratification range

Referring to the current study findings and established clinical risk reference ranges for cardiac biomarkers, our primary analysis involved stratifying the levels of NT-proBNP, hs-Troponin T, and hs-Troponin I based on their continuous values (18, 19, 38, 39). The cut points are divided into the following ranges: NT-proBNP: <125 pg/mL, 125 -<300 pg/mL, 300-<450 pg/mL and ≥450 pg/mL; hs-troponin T: <6 ng/L, 6-<14 ng/L and ≥14 ng/L; hs-troponin I (Abbott): male <6, femal <4 ng/L; male 6-12, femal 4-10 ng/L and male >12, female >10 ng/L. Elevated cardiac biomarkers are defined as increased levels of NT-proBNP (≥125 pg/mL), hs-Troponin T (≥6 ng/L), or hs-Troponin I (male ≥6, female ≥4 ng/L). Furthermore, we calculated the crude and age-adjusted prevalence of elevated cardiac biomarkers in participants with and without hyperuricemia.

### Hyperuricemia status

Serum uric acid level was measured with the Hitachi Model 704 multichannel analyzer (Boehringer Mannheim Diagnostics, Indianapolis, IN). Each participant was divided into groups with and without hyperuricemia based on their uric acid level. Based on the impact of drug use on individual serum uric acid levels, we selected uric acid-lowering or anti-gout medications (including Allopurinol, Probenecid) based on reports of prescribed medications from all participants. Hyperuricemia is defined as a condition in which the serum uric acid level exceeds 420 umol/L in males or 360 umol/L in females, or when undergoing treatment with uric acid-lowering or anti-gout medications (20, 21).

### Mortality

Data on mortality can be obtained from the mortality file published by the NCHS (http://www.cdc.gov/nchs/data-linkage/mortality.htm), where the personal identifier of each participant in the NHANES is linked to the corresponding death certificate records in the National Mortality Index (NDI). The follow-up time continued through December 31, 2019, and the precise cause of death was recorded using the International Classification of Diseases (ICD) 10th Revision, codes I00-I99. Cardiovascular death in this study was defined as any mortality related to cardiac or cerebrovascular disease.

### Other variable definitions

Participants reported personal information including age, gender, race and poverty income ratio (PIR) to well-trained interviewers during the family interview questionnaire. Blood pressure was measured three consecutive times in the right arm using a Mercury sphygmomanometer calibrated by Bowman meters, with a 30s interval between every measurement. Hypertension was defined as the average systolic blood pressure ≥140 mmHg or the average diastolic blood pressure ≥90 mmHg. The body mass index was calculated based on height and weight, and the formula was: (Kg)/(m^2^). Participants’ smoking status was evaluated through a detailed questionnaire survey and divided into three categories according to different standards subsequently: never, current and former. Drinking status was assessed based on questionnaire surveys and follow-up, and classified according to the following criteria into never drinking (had at most 12 alcohol drinks/lifetime), drinking occasional or a lot (had at least 12 alcohol drinks/lifetime or 12 alcohol drinks/1 year). Physical activity is based on the participants’ physical activity questionnaire, according to whether they had engaged in vigorous-intensity sports, fitness, or recreational activities for at least 10 min in the past month, resulting in a significant increase in breathing or heart rate; and whether they were involved in moderate-intensity sports, fitness or recreational activities cause small increases in breathing or heart rate and was done for at least 10 min continuously in a row to divide. Total cholesterol, high density lipoprotein cholesterol and triglycerides in blood samples were measured with a Hitachi Model 704 multichannel analyzer. Dyslipidemia is defined as total cholesterol ≥240 mg/dL, or triglyceride ≥150 mg/dL, or high-density lipoprotein cholesterol <40 mg/dL. Glycated hemoglobin (HbA1c) was measured using high-performance liquid chromatography. Diabetes was defined as self-reported diabetes diagnosed by doctors or HbA1c ≥6.5%. The renal function of participants was evaluated by the CKD-EPI equation, and individuals with severe renal insufficiency (eGFR <15 ml/min/1.73m^2^) were excluded from the study. The data on medications use was obtained from prescription reports, defined as the use of medications that directly or indirectly promote uric acid excretion within the past month. Specifically, this includes: allopurinol, probenecid, atorvastatin, fenofibrate, losartan, or Allisartan.

### Statistical analysis

According to hyperuricemia status, we examined the clinical characteristics of sociodemographic and cardiovascular risk factors among US adults. Normally distributed continuous variables were expressed as weighted mean ± SD, and analysis of variance was used to compare between groups; while skewed distributed were expressed as weighted median (M), with interquartile ranges (IQR) described the degree of dispersion, and the Kruskal Wallis rank-sum test was applied to compare between groups. Categorical data were described as proportions, and comparison between groups was performed using the Chi-square test or Fisher’s exact test. With attention to hyperuricemia status, we calculated the crude prevalence of elevated cardiac biomarkers among participants with and without hyperuricemia. As advised, we standardized age to further examine the age-adjusted prevalence of elevated cardiac biomarkers, using the age distribution of the US adult population in 2000 as a reference (22).

The research analysis was primarily divided into two parts: cross-sectional study was used to evaluate the correlation between elevated cardiac biomarkers and hyperuricemia; Retrospective analysis was used to evaluate the relationship between elevated cardiac biomarkers and long-term death in people with different hyperuricemia status. In the cross-sectional analysis, in order to examine the relationship between cardiac biomarkers and hyperuricemia, we developed multivariable logistic regression models, with all final models (model 3) adjusted for age, gender, race, PIR, hypertension, smoking status, alcohol, BMI, dyslipidemia, HbA1c, eGFR, medications use, physical activity and diabetes. To better characterize the continuing associations between NT-proBNP, hs-Troponin T and hs-Troponin I with hyperuricemia, we modeled each biomarker using restricted cubic splines, with 4 knots located at the 5th, 35th, 65th, and 95th percentiles, respectively. For the sensitivity analysis, we used the clinical cut-off point for elevated cardiac biomarkers as the limit to assess the association between elevated cardiac biomarkers and hyperuricemia risk among different age, gender, BMI, hypertension, smoking status, alcohol, medications use, physical activity, diabetes and dyslipidemia subgroups.

In the retrospective analysis, we used multivariate Cox regression model and Kaplan-Meier curve to examine the association between elevated cardiac biomarkers and all-cause mortality, and risk competition model and Cumulative Incidence Function (CIF) to examine the association between elevated cardiac biomarkers and cardiovascular mortality. Given the disparity in sampling probabilities across populations due to the oversampling of NHANES in certain subgroups, our analysis incorporated the recommended sampling weights to obtain unbiased estimates representative of the civilian.

All analyses were performed using the statistical package R (http://www.r-project.org; version 4.2.3), a *P*-value of <0.05 (double) was considered as statistically significant.

## Results

Among US adults without a history of cardiovascular disease, the prevalence of hyperuricemia was 16.35% from 1999 to 2004. Compared to those without hyperuricemia, participants with hyperuricemia tended to be older, with a higher proportion of males, regular smokers and medications use, and possess a higher body mass index, while displaying lower physical activity levels. In terms of cardiovascular risk factors, participants with hyperuricemia frequently exhibit a background of hypertension and diabetes, higher levels of dyslipidemia, glycated hemoglobin, and impaired renal function (**Table 1**).

**Table 1:** Characteristics of US adults without known cardiovascular disease by hyperuricemia status, NHANES 1999 to 2004.

Characteristic	All participants	No hyperuricemia	Hyperuricemia	*P*
n	6,439	N=5,395	N=1,044	
Age, y	44.3 (43.7,45.0)	43.7 (43.0,44.3)	47.7 (46.6,8.9)	<0.001
Gender, %				<0.001
Male	48.2	45.5	62.1	
Female	51.8	54.5	37.9	
Race and ethnicity, %				<0.001
Mexican American	7.34	7.88	4.58	
Other Hispanic	5.56	5.83	4.17	
Non-Hispanic White	74.57	74.12	76.91	
Non-Hispanic Black	8.56	8.44	9.18	
Other Race	3.96	3.73	5.15	
PIR	3.08 (2.96, 3.21)	3.08 (2.95, 3.20)	3.10 (2.93, 3.27)	0.722
Hypertension, %				<0.001
No	84.30	86.00	75.63	
Yes	15.70	14.00	24.37	
Body mass index, kg/m2	27.9 (27.7,28.2)	27.3 (27.1,27.6)	31.1 (30.5,31.7)	<0.001
Smoking status, %				0.001
Never	50.56	51.20	47.25	
Current	25.28	25.67	23.27	
Former	24.16	23.12	29.48	
Alcohol, %				0.161
Never	11.50	11.21	13.00	
Occasionally/a lot	88.50	88.79	87.00	
Dyslipidemia, %				<0.001
No	61.50	65.05	43.36	
Yes	38.50	34.95	56.64	
HbA1c, (%)	5.4 (5.4,5.4)	5.4 (5.3,5.4)	5.5 (5.5,5.6)	<0.001
eGFR_CKD–EPI_ (ml/min/1.73m2)	99.1 (98.0,100.2)	100.7 (99.7,101.8)	90.7 (88.8,92.5)	
Diabetes, %				0.001
No	94.93	95.27	93.17	
Yes	5.07	4.73	6.83	
Medications use, %				<0.001
No	98.60	99.13	95.82	
Yes	1.40	0.87	4.18	
Physical activity (%)				<0.001
Vigorous	37.74	38.85	32.05	
Moderate	29.14	29.01	29.83	
Lacking	33.12	32.14	38.12	

Continuous variables are described by mean ± standard deviation or median (quartile spacing); categorical variables are described as proportions. All means and proportions are weighted estimates. NHANES, National Health and Nutrition Examination Survey; PIR, poverty income ratio; HbA1c, glycated hemoglobin; eGFR, estimated glomerular filtration rate.

**Figure 2:**
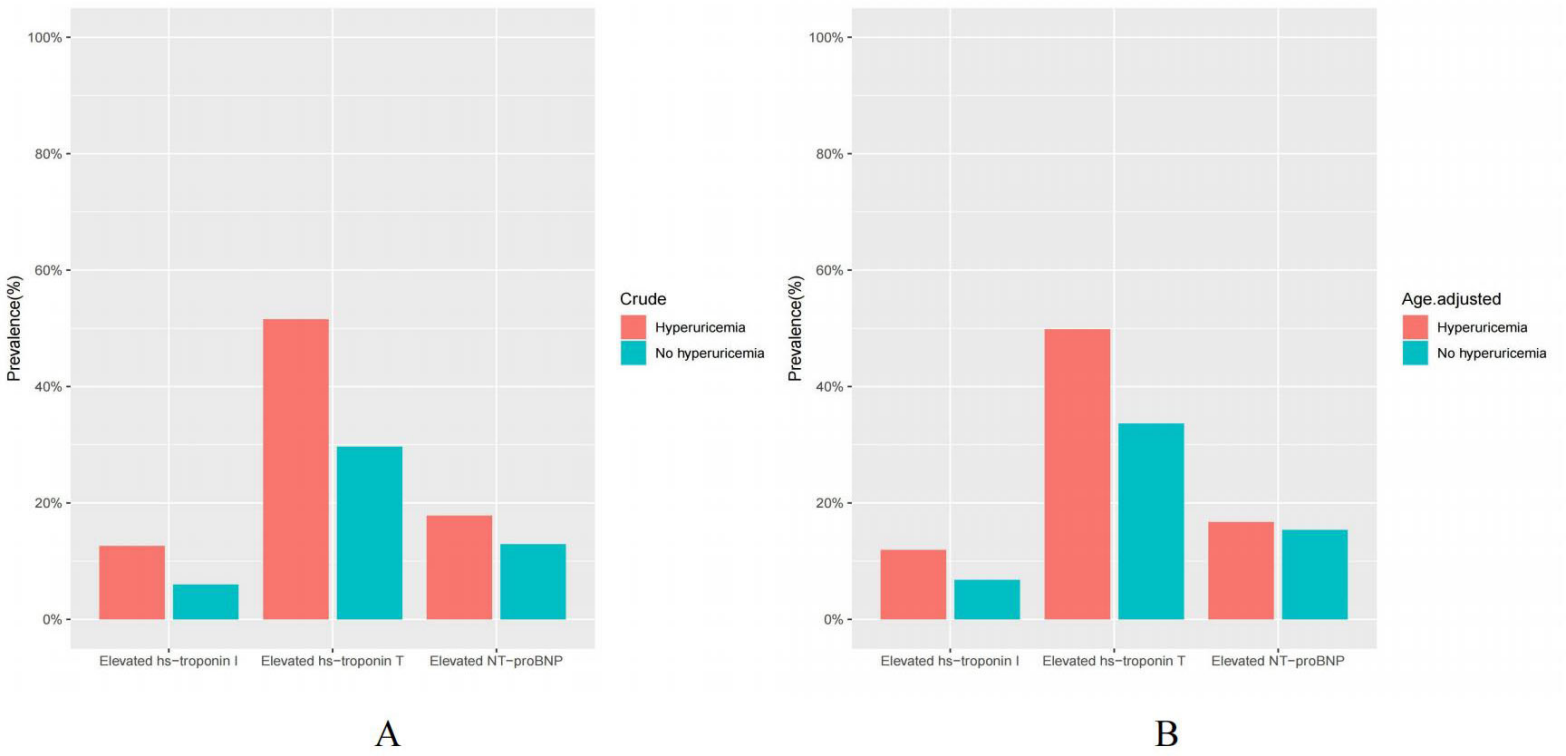
**(A, B)** Crude and age-adjusted prevalence of elevated cardiac biomarkers in US adults by hyperuricemia status, NHANES 1999 to 2004. Elevated NT-proBNP (≥125 pg/mL); Elevated hs-troponin T (≥6 ng/L); Elevated hs-troponin I (male ≥6, female ≥4 ng/L).

Among US adults without known cardiovascular disease but with hyperuricemia, the crude prevalence of elevated NT-proBNP (≥125 pg/mL), hs-troponin T (≥6 ng/L), and hs-troponin I (male ≥6, female ≥4 ng/L) was 17.83%, 51.56%, 12.63%, respectively (**Table 2**, **Figure 2A**). Upon conducting standardization based on age, the age-adjusted prevalence of elevated cardiac biomarkers were found to be 16.70%, 49.80%, 11.91%, respectively (**Table 2**, **Figure 2B**). Furthermore, the prevalence of elevated hs-troponin I in people with hyperuricemia is roughly twice (11.91% versus 6.75%) as high as those without hyperuricemia, a distinction that is notably remarkable.

**Table 2:** Crude and age-adjusted prevalence of elevated cardiac biomarkers in US adults by hyperuricemia status, NHANES 1999 to 2004.

Prevalence	No hyperuricemia	hyperuricemia
Crude		
elevated NT-proBNP	12.90%	17.83%
elevated hs-troponin T	29.68%	51.56%
elevated hs-troponin I	5.96%	12.63%
Age-ajusted		
elevated NT-proBNP	15.36%	16.70%
elevated hs-troponin T	33.61%	49.80%
elevated hs-troponin I	6.75%	11.91%

**Figure 3:**
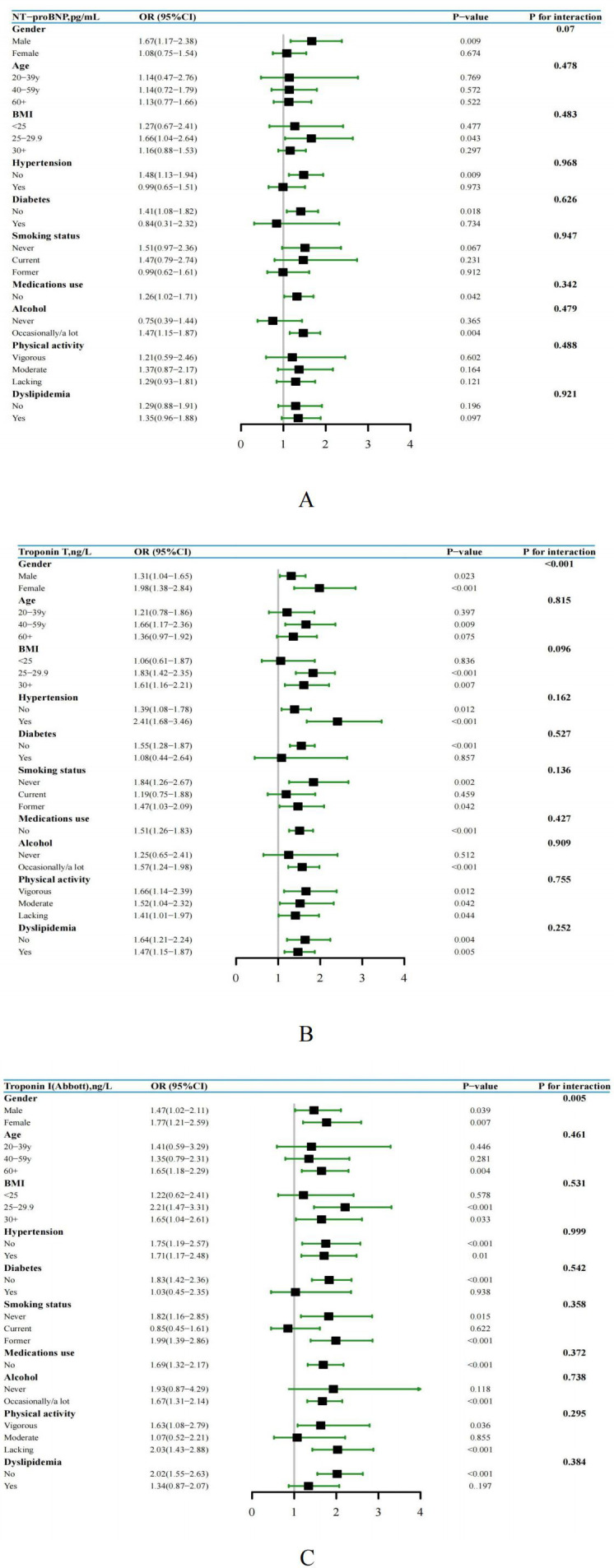
**(A-C)** Subgroup analysis and forest plot of NT-proBNP, hs-Troponin T, and hs-Troponin I with hyperuricemia by gender, age, BMI, hypertension, smoking status, alcohol, medications use, physical activity, diabetes and dyslipidemia, US adults without known cardiovascular disease, NHANES 1999 to 2004.

After controlling for demographic characteristics including age, gender, race and PIR, multivariable logistic regression analysis revealed a statistically significant association between elevated levels of NT-proBNP, hs-troponin T, and hs-troponin I and hyperuricemia. In addition, different clinical categories observed noticeable differences in grades on the same cardiac biomarker. After further adjusting for cardiovascular risk factors, the correlation between elevated cardiac biomarkers and hyperuricemia was attenuated but remained statistically significant (**Table 3**). Furthermore, a subgroup analysis encompassing age, gender, BMI, hypertension, smoking status, alcohol, medications use, physical activity, diabetes and dyslipidemia was performed. Specifically, the results indicated that in different subgroups, the significant correlation between elevated levels of NT-proBNP, hs-troponin T, and hs-troponin I with hyperuricemia persisted, and the results were consistent with the overall population trend. Furthermore, through interaction testing, apart from gender, we did not observe significant effects of age, BMI, hypertension, smoking status, alcohol, medications use, physical activity, diabetes and dyslipidemia on the associations between elevated cardiac biomarkers and hyperuricemia (**Figures 3A–C**).

**Table 3:** Adjusted association of NT-proBNP, hs-Troponin T, and hs-Troponin I with hyperuricemia, US adults without known cardiovascular disease, NHANES 1999 to 2004.

Clinical cut point	Hyperuricemia, OR (95% CI)		
	Model 1: unadjusted	Model 2: adjusted	Model 3: adjusted
NT-proBNP, pg/mL			
<125	1 (reference)	1 (reference)	1 (reference)
125–<300	1.16 (0.92, 1.46)	1.15 (0.88, 1.51)	1.15 (0.86, 1.52)
300–<450	1.90 (0.98, 3.70)	1.66 (0.83, 3.29)	1.49 (0.77, 2.92)
≥450	3.11 (2.20, 4.38)	2.43 (1.58, 3.71)	2.41 (1.51, 3.85)
*p* for trend	< 0.001	< 0.001	0.002
hs-troponin T, ng/L			
<6	1 (reference)	1 (reference)	1 (reference)
6–<14	2.34 (2.04, 2.69)	1.85 (1.54, 2.22)	1.50 (1.26, 1.79)
≥14	3.71 (2.82, 4.86)	2.70 (1.89, 3.85)	2.04 (1.43, 2.93)
*p* for trend	< 0.001	< 0.001	< 0.001
hs-troponin I, ng/L			
M, <6; F, <4	1 (reference)	1 (reference)	1 (reference)
M, 6–12; F, 4–10	2.43 (1.90, 3.09)	1.99 (1.56, 2.54)	1.77 (1.33, 2.35)
M, >12; F, >10	1.90 (1.32, 2.75)	1.58 (1.06, 2.36)	1.50 (1.02, 2.26)
*p* for trend	< 0.001	< 0.001	< 0.001

Model 1 was unadjusted.

Model 2 was adjusted for age, gender, race and ethnicity and PIR.

Model 3 was adjusted for age, gender, race and ethnicity, PIR, smoking status, alcohol, hypertension, body mass index, dyslipidemia, HbA1c, eGFR_CKD–EPI_, diabetes, medications use, physical activity.

OR, odds ratio; M, male; F, female.

**Figure 4:**
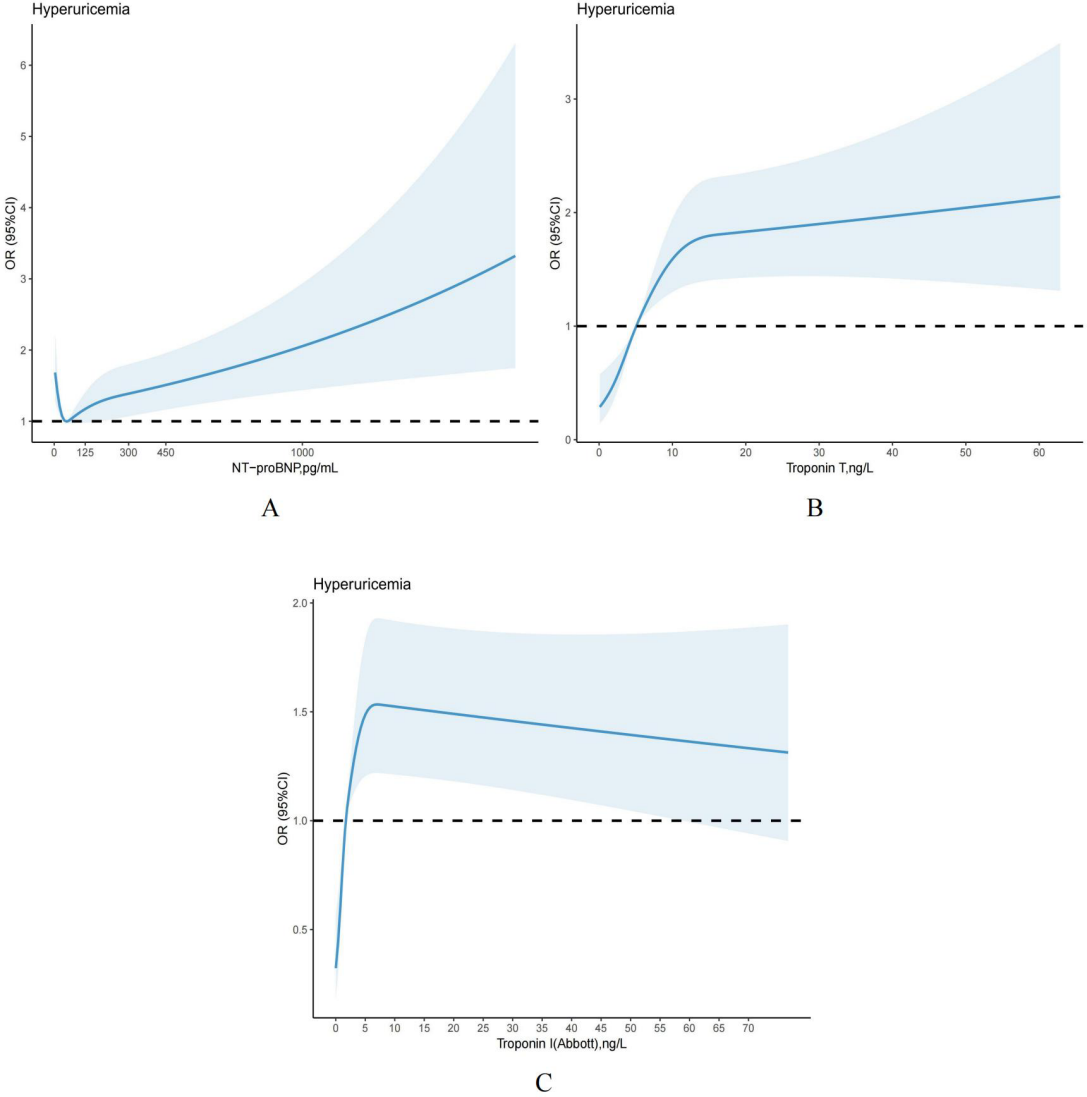
**(A-C)** Restricted cubic splines models demonstrating continuous association of cardiac biomarkers with hyperuricemia, NHANES 1999 to 2004. Models were adjusted for age, gender, race and ethnicity, PIR, smoking status, alcohol, hypertension, body mass index, dyslipidemia, HbA1c, eGFR_CKD–EPI_, diabetes, medications use, physical activity.

The restricted cubic spline is used as a visual tool to model the relationship between each elevated cardiac biomarker and hyperuricemia. Based on the findings, NT-proBNP and hs-troponin T showed a significant association with hyperuricemia, with a gradual increase in the risk of developing hyperuricemia (**Figures 4A, B**). Furthermore, we observed that as the level of hs-troponin I increased, the risk of hyperuricemia initially increased and then decreased (**Figure 4C**), which was aligned with the outcomes of multiple regression analysis, indicating that elevated hs-troponin I may have a segmented effect on hyperuricemia.

**Figure 5:**
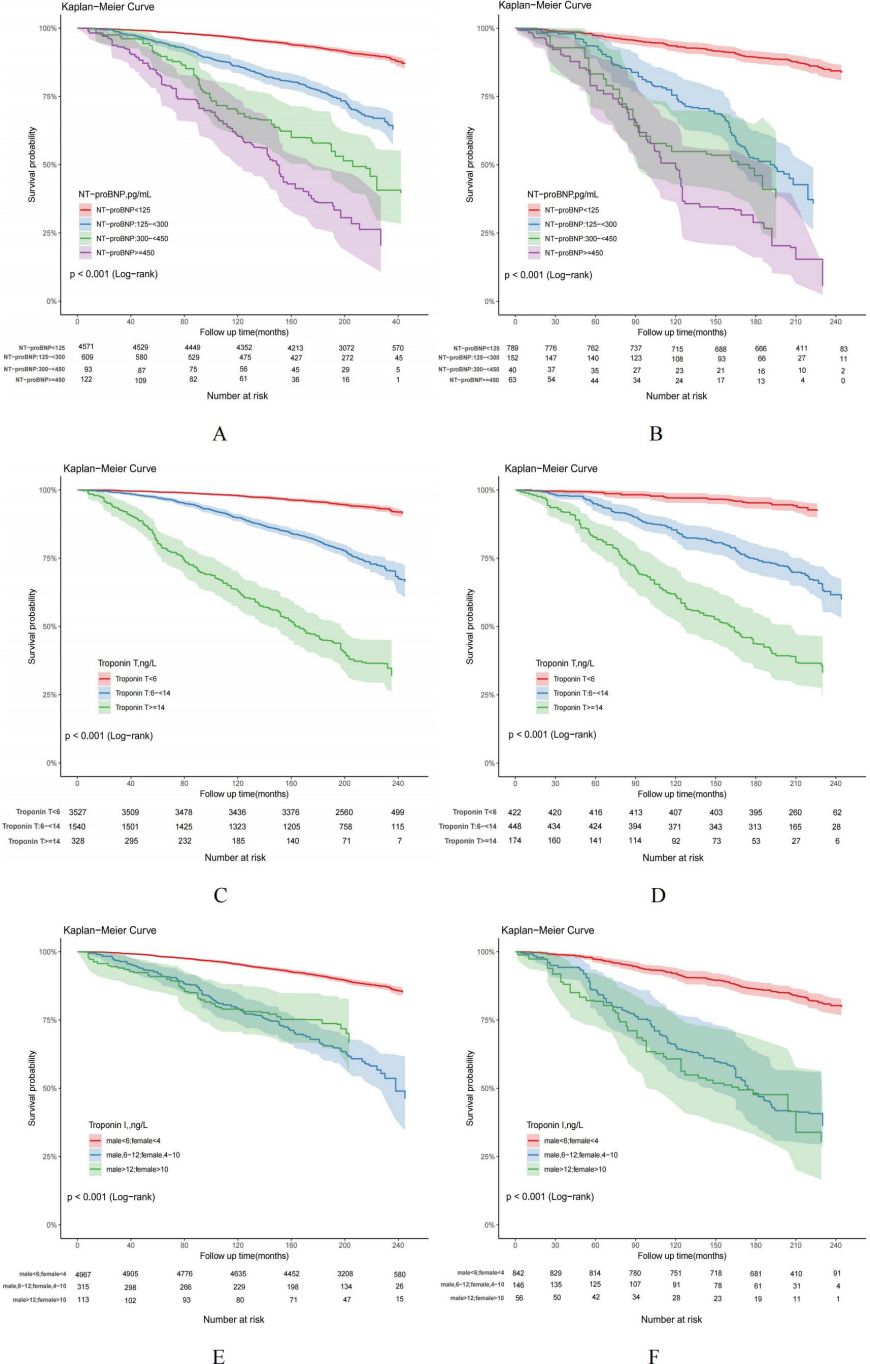
**(A–F)** Kaplan-Meier curves for all-cause mortality in US adults by hyperuricemia status, NHANES 1999-2004. US adults without hyperuricemia: **(A, C, E)**; US adults with hyperuricemia: **(B, D, F)**.

**Figure 6:**
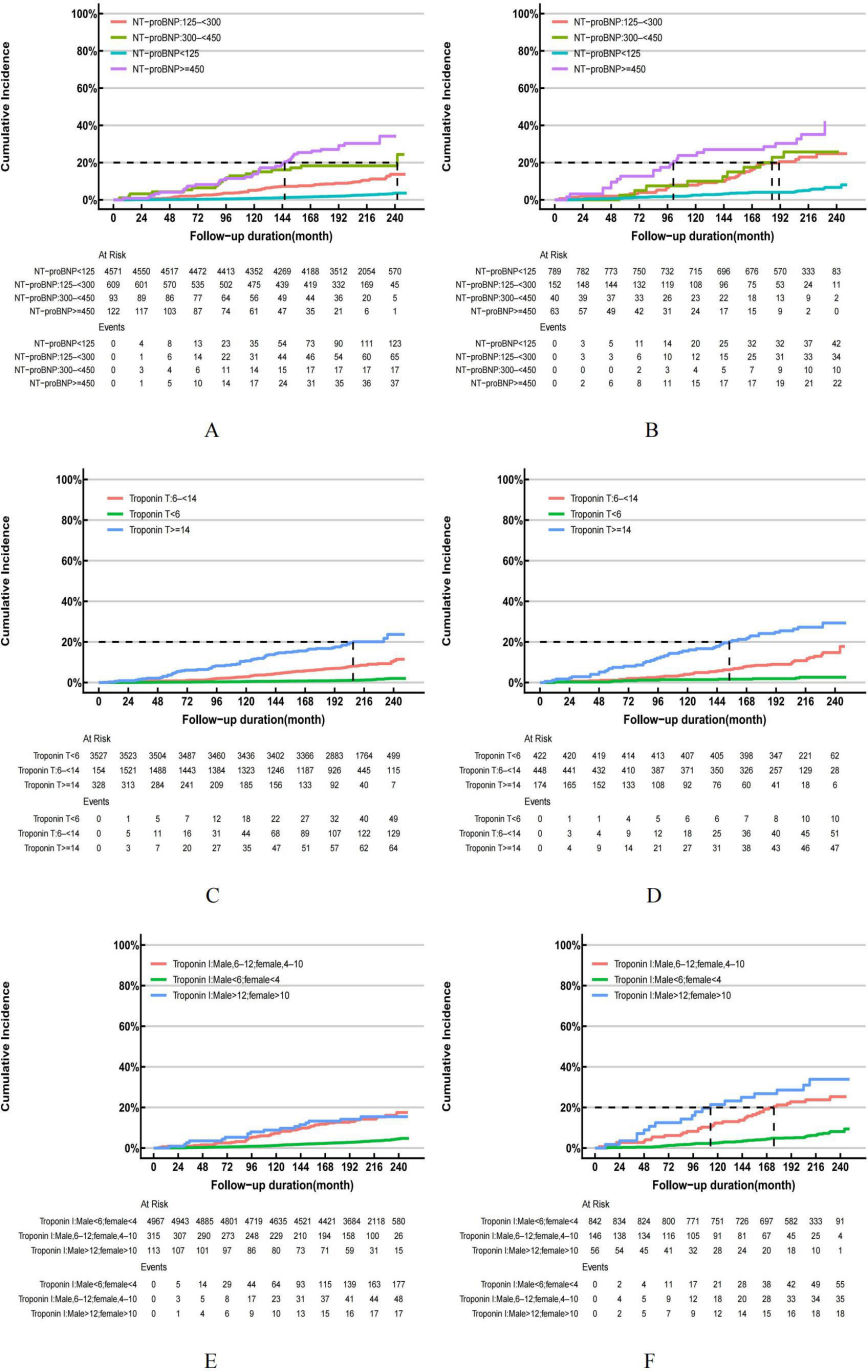
**(A–F)** Cumulative Incidence Function (CIF) for cardiovascular mortality in US adults by hyperuricemia status, NHANES 1999-2004. US adults without hyperuricemia: **(A, C, E)**; US adults with hyperuricemia: **(B, D, F)**.

Based on the follow-up data, it can be observed that the overall mortality rate among individuals without hyperuricemia was recorded at 18.74%, whereas adults with hyperuricemia experienced a higher cumulative mortality rate of 32.66%. Cox regression and risk competition model findings indicate that with or without hyperuricemia, elevated NT-proBNP, hs-troponinT and hs-troponinI were each significantly positively associated with the cumulative incidence of all-cause (**Figures 5A–F**) and cardiovascular mortality (**Figures 6A–F**). Compared to those with solely elevated cardiac biomarkers, adults with hyperuricemia and elevated cardiac biomarkers faced the highest risk of all-cause and cardiovascular mortality (**Table 4**), with the exception of the association between elevated hs-troponin I and all-cause mortality.

**Table 4:** Associations* (Hazard Ratio [95% CI]) between NT-proBNP, hs-Troponin T, and hs-Troponin I and mortality (all-cause and CVD mortality) in different hyperuricemia status, NHANES 1999-2004.

Clinical cut point	All-Cause Mortality		Cardiovascular Disease Mortality	
	Hyperuricemia status		Hyperuricemia status	
	No	Yes	No	Yes
NT-proBNP, pg/Ml				
<125	1 (Ref)	1 (Ref)	1 (Ref)	1 (Ref)
125–<300	1.39 (1.14-1.71)	1.83 (1.25-2.69)	1.27 (0.89-1.82)	1.84 (1.07-3.15)
300–<450	2.05 (1.48-2.84)	1.95 (1.30-2.92)	1.70 (0.95-3.04)	1.77 (0.83-3.76)
≥450	2.09 (1.52-2.89)	3.49 (2.25-5.41)	2.23 (1.41-3.52)	2.25 (1.15-4.41)
hs-troponin T, ng/L				
<6	1 (Ref)	1 (Ref)	1 (Ref)	1 (Ref)
6–<14	1.31 (1.06-1.63)	2.08 (1.26-3.44)	1.47 (0.97-2.22)	1.55 (0.69-3.47)
≥14	2.18 (1.62-2.93)	3.46 (2.04-5.85)	1.75 (1.04-2.93)	1.90 (0.74-4.90)
hs-troponin I, ng/L				
M, <6; F, <4	1 (Ref)	1 (Ref)	1 (Ref)	1 (Ref)
M, 6–12; F, 4–10	1.47 (1.19-1.82)	1.59 (1.21-2.09)	1.39 (0.98-1.97)	1.48 (1.05-2.49)
M, >12; F, >10	1.78 (1.14-2.77)	1.72 (1.07-2.76)	1.63 (1.03-2.89)	2.28 (1.09-4.77)

*adjusted for age, gender, race and ethnicity, PIR, smoking status, alcohol, hypertension, body mass index, dyslipidemia, HbA1c, eGFR_CKD–EPI_, diabetes, medications use, physical activity.

M, male; F, female.

## Discussion

Our study revealed a notable disparity in the occurrence of elevated cardiac biomarkers between individuals with and without hyperuricemia among US adults without known cardiovascular disease. More precisely, nearly half of adult patients with hyperuricemia had elevated levels of hs-troponin T, 16.70% had elevated levels of NT-proBNP, and 11.91% had elevated levels of hs-troponin I. Additionally, elevated levels of all three cardiac markers are strongly correlated with hyperuricemia. Clearly, these observations indicate that elevated cardiac biomarkers pose a huge burden on hyperuricemia risk in the general population. Simultaneously, it further highlights the significant role of elevated cardiac biomarkers in the timely identification of risk factors in individuals with hyperuricemia.

In contrast to hypertension, diabetes, and other risk factors, hyperuricemia possesses a certain element of obscurity. Prior to the onset of gout, the health risks associated with hyperuricemia tend to be overlooked. However, over the past few decades, there has been a progressive rise in the prevalence of hyperuricemia among the middle-aged and elderly demographic. Additionally, it has been substantiated that hyperuricemia is closely linked to the emergence and progression of metabolic syndrome, cardiovascular disease, and chronic kidney disease (23, 24). Through long-term follow-up data analysis, our study found that individuals afflicted with hyperuricemia, in conjunction with elevated cardiac biomarkers, faced a higher risk of all-cause and cardiovascular mortality than those with elevated cardiac biomarkers alone. At this point, prior research has demonstrated that elevated cardiac biomarkers are independent risk factors for increased all-cause and cardiovascular mortality in the general population (18). Furthermore, chronic hyperuricemia is associated with an elevated risk of cardiovascular diseases and long-term mortality in the population. Therefore, our observation that individuals with elevated cardiac biomarkers combined with hyperuricemia present the highest mortality risk should be considered reasonable and can be interpreted as the cumulative effect of the interplay between the two factors. Hence, by examining the association between elevated cardiac biomarkers and the risk of hyperuricemia, we can identify individuals at high risk early and implement precise interventions for risk stratification, which holds significant implications for mitigating general population health risks.

As we all know, NT-proBNP is a recognized biomarker in clinical diagnosis, serves as an effective diagnostic tool for identifying heart failure (8, 9, 25). It is a natriuretic peptide hormone, which is released by cardiomyocytes when ventricular wall tension increases but lacks biological activity (26). Previous studies have found that serum uric acid levels and elevated NT-proBNP are correlated in individuals diagnosed with unstable angina pectoris or without significant heart failure (27, 28). Additionally, hyperuricemia has been proven as a reliable independent prognosticator for unfavorable outcomes, including all-cause mortality, in patients with chronic heart failure (29, 30). Nevertheless, it is important to note that these researchers have predominantly focused on individuals with prevalent cardiovascular disease, and lack estimates of the level of a larger population. Therefore, our study aimed to evaluate the effects of elevated NT-proBNP on the risk of hyperuricemia and long-term mortality among US adults without prevalent cardiovascular disease, and performed further subgroup analyses to determine the risk estimates for different populations.

Furthermore, previous studies have indicated the association between hs-troponin T and various heart and non-heart diseases, such as stroke, asymptomatic cerebral infarction, and advanced kidney disease (31–34). And elevated hs-troponin T levels have also been shown associated with an increased risk of cardiovascular events in individuals with type 2 diabetes without cardiovascular disease (35–37). To our knowledge, the relationship between hs-troponin T, hs-troponin I and hyperuricemia has not yet been reported in adults without prevalent cardiovascular disease. According to our study, elevated levels of hs-troponin T or hs-troponin I were correlated with an increased risk of all-cause and cardiovascular mortality in individuals with hyperuricemia. Even after adjusting for traditional cardiovascular risk factors, this elevated risk persisted and also existed in individuals without hyperuricemia. Thus, in conjunction with the examination of NT-proBNP and hyperuricemia, these findings propose that routine detection of cardiac biomarkers among adults without prevalent cardiovascular diseases could serve as an effective strategy for preemptive early risk mitigation. But this requires further research in the future to assess its advantages.

Among US adults with hyperuricemia or non-hyperuricemia, our study showed that the crude prevalence and age-adjusted prevalence of hs-troponin T was notably higher than that of hs-troponin I, which was consistent with previous findings conducted on individuals with diabetes or peripheral arterial disease (38, 39). However, it may not be accurate to conclude that hs-troponin T is more sensitive and accurate in identifying high-risk cardiovascular populations compared to hs-troponin I. Generally, an elevated high-sensitivity troponin level indicates myocardial injury events. According to theory, manufacturers establish the cutoff for elevated hs-troponin by identifying the 99th percentile concentration in healthy reference samples (i.e., the prevalence of the increase of hs- troponin in healthy reference population is close to 1%). Based on this, using the manufacturer’s recommended cutoff for hs-troponin T, approximately half of the population shows elevated hs-troponin T levels. Therefore, a reasonable explanation could be that the 99% upper limit reference value of health definition in the health reference sample is set too strictly. In contrast, diagnosing myocardial injury with the elevated threshold of hs-troponin I results in a lower disease rate, which seems to be more in line with the real situation in the prevention population. However, despite the significant difference in positive disease rates between the two, they still provide additional independent information on all-cause mortality and cardiovascular death risks in the general adult population, and it is also helpful to evaluate the risk stratification of high-risk groups more accurately in the process of clinical diagnosis and treatment.

At present, the precise biological mechanism underlying the correlation between NT-proBNP, hs-troponin T, hs-troponin I, and hyperuricemia remains unclear. We can assume that NT-proBNP is released from cardiomyocytes in response to myocardial stretching or tissue hypoxia, and hypoxia might elevate serum uric acid levels through the activation of xanthine oxidoreductase (40). These assumptions need to be further explored in future studies.

Our research is groundbreaking as it is the initial study to employ a nationally representative sample to evaluate the association between elevated cardiac biomarkers and hyperuricemia in adults without prevalent cardiovascular diseases in the US. Furthermore, by conducting long-term follow-up studies, we have determined the influence of elevated cardiac biomarkers on long-term mortality risk in different populations. These results provide evidence that support early risk stratification and intervention in adults without cardiovascular disease through elevated cardiac biomarkers. Thus, this is undoubtedly a significant advantage of our research.

Our research also has several limitations. First, we only examined the relationship between single biomarkers, such as NT-proBNP, hs-troponin T and hs-troponin I, and hyperuricemia, without evaluating the combined effect of multiple biomarkers. Second, the medical history of cardiovascular disease in adults is based on self-diagnostic reports from the past, which may introduce certain errors due to subjective factors among the participants. Finally, our study results cannot be compared with other studies’ conclusions to determine whether the conclusions are consistent or contradictory due to the lack of previous research on the relationship between cardiac biomarkers and hyperuricemia.

In conclusion, our findings suggest that elevated cardiac biomarkers pose a high burden on the risk of hyperuricemia in the general population without prevalent cardiovascular disease, and further provide important information regarding future mortality risk in these populations. Routine testing of cardiac biomarkers in high-risk populations proves advantageous for early risk identification and prognostic assessment.

## Data Availability

The datasets presented in this study can be found in online repositories. The names of the repository/repositories and accession number(s) can be found in the article/supplementary material.
